# 

**Published:** 1875-04-01

**Authors:** 


No. VII. APRIL 1, 1875. Vol. XVI.

### THE MEDICAL EXAMINER:

A

### Semi-Monthly Journal of Medical Sciences.

EDITED BY

N. S. DAVIS, M. D., and F. H. DAVIS, M. D.

*Subscription Price, Three Dollars per Annum, in advance.*

*Single Numbers, Fifteen Cents.*

CHICAGO.

PUBLISHED AT 65 E. RANDOLPH STREET.

